# What and how: doing good research with young people, digital intimacies, and relationships and sex education

**DOI:** 10.1080/14681811.2020.1732337

**Published:** 2020-03-17

**Authors:** Rachel H. Scott, Clarissa Smith, Eleanor Formby, Alison Hadley, Lisa Hallgarten, Alice Hoyle, Cicely Marston, Alan McKee, Dimitrios Tourountsis

**Affiliations:** a Centre for Research in Media and Cultural Studies, Faculty of Arts and Creative Industries, University of Sunderland, Sunderland, UK; b Sheffield Institute of Education, Sheffield Hallam University, Sheffield, UK; c Institute for Health Research, University of Bedfordshire, Luton, UK; d Brook, London, UK; e Independent Consultant, Bath, UK; f Department of Public Health, Environments and Society, Faculty of Public Health and Policy, London School of Hygiene & Tropical Medicine, London, UK; g Faculty of Arts and Social Sciences, University of Technology Sydney, Sydney, Australia

**Keywords:** Relationships and sex education, young people, research methods, technology, digital intimacies

## Abstract

As part of a project funded by the Wellcome Trust, we held a one-day symposium, bringing together researchers, practitioners, and policymakers, to discuss priorities for research on relationships and sex education (RSE) in a world where young people increasingly live, experience, and augment their relationships (whether sexual or not) within digital spaces. The introduction of statutory RSE in schools in England highlights the need to focus on improving understandings of young people and digital intimacies for its own sake, and to inform the development of learning resources. We call for more research that puts young people at its centre; foregrounds inclusivity; and allows a nuanced discussion of pleasures, harms, risks, and rewards, which can be used by those working with young people and those developing policy. Generating such research is likely to be facilitated by participation, collaboration, and communication with beneficiaries, between disciplines and across sectors. Taking such an approach, academic researchers, practitioners, and policymakers agree that we need a better understanding of RSE’s place in lifelong learning, which seeks to understand the needs of particular groups, is concerned with non-sexual relationships, and does not see digital intimacies as disconnected from offline everyday ‘reality’.

## Introduction

Adults worry about young people and their use of technology. Social media and the Internet, and sexual content, in particular, are often blamed for a range of problematic outcomes among young people – from poor body image and mental health issues to bullying and coercive sexual attitudes – yet the evidence base for these claims is not always robust (C. Marston 2018). Alongside claims of effects, the research used to inform public health interventions has often struggled to keep step with technology and the lived experience of young people (Thomson, Berriman, and Bragg 2018). This has profound implications for how young people are supported to develop the skills and confidence to navigate relationships and secure their health and wellbeing.

Digital media have become central to how friendships, family connections, and romantic and sexual relationships are cultivated, developed, and maintained. All forms of intimacy are, and have always been, in some way mediated (Attwood 2017), in that they ‘require a medium through which intimate relations can be established between the subject and the other’ (Attwood, Hakim, and Winch 2017). Even so, intimacies mediated through digital technologies are still considered relatively new, and research as well as policy and practice responses are constantly playing catch-up – particularly given rapid and continuing technological developments and the speed with which young people take up and abandon particular platforms. The terms ‘mediated intimacy’ and ‘digital intimacy’ are useful in this context first, to emphasise the educative importance of media forms such as sex and relationship advice and ‘agony aunts’ in the development of sexual knowledge and the management of sexual health (Barker, Gill, and Harvey 2018); and second, to denote the ways in which intimacy is now formed through connections between people within networked environments using devices, apps, and platforms (Baym 2015; Chambers 2013; Paasonen 2018). These dual functions of mediated intimacies extend into the roles that digital media play in relation to relationships and sex education (RSE) – they are a medium through which intimacies are formed and negotiated between young people, and also through which education about intimacies can be developed and shared, both through formal campaigns and peer-to-peer information sharing. A key feature of digital media in this context is that they allow young people to be both the consumers and producers of ‘education’.

The ways in which digital media have become integral to different forms of intimacy have significant implications for our understandings of what constitutes healthy sexuality, yet there is no agreement about how and at what age parents and professionals working with young people should address questions relating to digitally mediated sexual content and experiences. Relationships education at primary school level and relationships and sex education at secondary school level will become statutory in all schools in England from September 2020. Although young people’s satisfaction with their school-based RSE has increased (Ofsted 2013; Sex Education Forum 2018a), provision in England is patchy, with variability in quality and content (Formby et al. 2011; Ofsted 2013). Teachers report sexually explicit online content as one of the topics they feel least confident teaching about (Sex Education Forum 2018b). Topics such as pornography, ‘sexting’^1^ and the use of online media are included in the new guidance (Department for Education 2019). This guidance, however, is deliberately non-prescriptive so implementation is likely to vary considerably between schools with respect to the extent to which topics will be addressed, at what age, and how. Many schools will be looking for resources and interventions that are high-quality, relevant, engaging and evidence-based. RSE also takes place outside of school settings, via websites like BISH, Scarleteen, Brook, and The Mix and through social media ‘influencers’, and in clinical settings and youth projects. Understanding how young people engage with digital intimacies will be crucial to informing the development of high-quality RSE across settings.

We use the term ‘digital intimacies’ to encompass a wide range of practices including sexually explicit image sharing; taking and sharing selfies; meeting sexual partners; communicating about sex and relationships; searching for information and advice; and creating, accessing, and circulating sexual content online, through social media and through apps. Digital intimacies also refer to how young people engage in communications via digital platforms/technologies to forge intimacy – the ‘kinds of connection that *impact* on people, and on which they depend for living’ (Berlant 1998, 284; original emphasis). Intimacy defines many forms of relationships – from familial through friendship to romantic and sexual. Digital spaces offer new ways to connect, and since digital technology is now so much part of the ‘woodwork’ of everyday life (Mosco 2004, 21), digital media offer connections and relationships that are as effective, complex, messy, sustaining and problematic as those forged offline, indeed offline, and online relationships may be so entangled as to be inseparable.

As part of the ‘Investigating mediated sex and young peoples’ health and well-being’ project funded by the Wellcome Trust (207971), we held a one-day workshop to discuss priorities for research on RSE in a world where young people increasingly live, experience, and augment their relationships (whether sexual or not) within digital spaces. The workshop involved practitioners and representatives from organisations working with young people; policymakers; and researchers with expertise in young people, sexual behaviour, sex education, digital media, porn studies, critical sociology, sexuality, and gender studies. Our aims were to discuss the future research agenda in this area and set priorities for research approaches and content. The day began with presentations from academics and practitioners, followed by small group discussions on specific topics that were then shared with the whole group, and finally a roundtable discussion (see online supplementary material for details of the workshop agenda).

This article outlines some of our key discussions and offers recommendations for research. These recommendations are not exhaustive, but provide a starting point based on dialogue and consensus from the workshop. The workshop focussed on areas where there was consensus across participants, so while there were few areas where contention or strong differences in opinion were voiced, there were some divergent views in terms of what research would have traction and be most useful, and participants highlighted the challenges inherent in working across disciplines and across sectors. We describe these in the relevant sections. Many themes that emerged during the workshop are relevant to research into sex and relationships more generally, so could be applied beyond the area of digital intimacies and in a range of settings. Furthermore, while our discussion was focused on RSE in the UK, in both school and non-school settings, many points also have relevance to comprehensive sexuality education elsewhere.

## Priority approaches to research with young people, digital intimacies and RSE

### Incorporate participation with stakeholders from the start

Where possible, research should be participatory throughout and involve relevant stakeholders in design, analysis, and dissemination. Participants must include young people themselves but might also include teachers or other stakeholders depending on the topic and research aims. The research questions should be derived at least in part from dialogue with stakeholders. Analysis can also be conducted in conjunction with stakeholders in different ways, including for instance discussion of emerging findings and incorporation of those discussions into ongoing analysis. Participatory research can be radical, and can be a deliberate way of trying to disrupt certain agendas although it can also be exploitative and self-serving if done in bad faith (Cooke and Kothari 2001).

Young people’s involvement in the research process is essential to achieve meaningful and relevant research that meets their needs and interrogates what they identify as risks, problems, mitigations, and solutions, particularly in the context of fast moving developments in the digital world and because there are many different platforms specific to young people’s age and stage of development. Albury et al. (2013) argue that young people do have a voice, and express it when asked, but may still not be listened to. Tokenistic consultation is unethical; it devalues young people’s contributions and wastes their time. Seeking out young people’s voices must take place through genuine dialogue. At a bare minimum young people can be invited to sit on advisory committees so their experiences inform the research (Albury et al. 2013), although it is important that occasions on which they are asked to contribute are not solely conducted on older adults’ terms (e.g. in locations not easily accessible to young people like hospital meeting rooms, or at inconvenient times like during school examinations). It is also important to pay attention to which young people are invited to participate – people in participatory processes can be unfairly expected to represent everyone in their category (Renedo, Komporozos-Athanasiou, and Marston 2018) and diversity of experience among young people needs to be acknowledged. Research focusing on digital media may facilitate young people’s participation, due to high rates of engagement in digital activities among this group, but researchers must also take care to enable participation of those who engage with less intensity or in more unusual ways, and those who are less represented online or to whom the online world is less accessible. Research with young people should also recognise that what people say is shaped by norms and their social environment; research that does give a platform to young people’s voices should seek to interrogate how dominant social norms and expectations shape the kinds of discourse young people have access to (see Smith and Attwood 2011). Examples of participatory research with young people, including some discussion of the challenges inherent in this work, include projects by Miles, Renedo, and Marston (2018); 2019), C. Marston (2004, 2005), K. Marston (2019), and Renold (Libby et al. with Renold 2018, Renold 2018).

### Collaborate across academic disciplines and knowledge-production sectors

The study of human sexualities and digital practices is complex and suited to a more interdisciplinary approach than has been common in health research. Collaboration between researchers from different disciplines and with different methodological expertises can help break out of disciplinary and methodological silos and generate enhanced understandings of how young people engage with digital intimacies and the main issues that concern them. Collaboration between researchers in, for example, media studies and public health present opportunities to understand the intersections between sex, technology, digital practices, health and wellbeing.

Collaboration across sectors can support practitioners to critically evaluate research evidence, conduct high-quality research, and ensure that resources and interventions are informed by up-to-date research, as well as increasing the depth, relevance and impact of research projects. Policymakers, practitioners and researchers have differing areas of skills, knowledge and influence, which can be leveraged at different stages of the research process. For example, practitioners (i.e. individuals that work directly with young people, such as health-care providers, youth, and social workers, teachers or other educators) have understandings of young people’s everyday lives and experiences that can provide an essential contribution to the development of research questions and the design of research projects. Policymakers can help inform research that responds meaningfully to current policy questions. Academic researchers who are willing to engage with non-academic stakeholders to understand what information they need, and the format in which they need it delivered, are more likely to produce relevant results and be able to frame them in ways that are understandable to non-specialists, which ultimately are more likely to be taken up outside academia.

The experiences of individuals working with young people are also useful in theory building, and collaboration between academics and practitioners, who each bring different expertises, can help build more comprehensive and relevant theoretical frameworks around research projects or interventions than either group working alone. Whilst researchers may be experts on study design, practitioners can provide invaluable insight into the practicalities of conducting research in a given setting with a given population, in terms of accessing participants and providing spaces in which research can take place, and insights to inform emerging analyses. Where practitioners conduct independent research, academic partners can provide methodological and technical expertise to support the research process and advise on what is feasible and could generate useful and valid findings.

Where appropriate, and with the correct training, practitioners may be involved in data collection, although ethical issues regarding moving from a practitioner to a researcher role, for example around obligations to report disclosures, must be considered. Practitioners are able to utilise existing trusted relationships with young people to help them engage comfortably and confidently in the research process, and good communication skills which have potential to gain more granular insights into young people’s feelings, attitudes, and behaviour and how these interplay with the wider context of their lives. They can bring creative approaches to engaging with young people in a research setting. Trained researchers and practitioners can work together to analyse data, for example by discussing preliminary findings, helping researchers to stay closer to the questions that are important to the research users as well as improve the insights from the research. At the dissemination stage, policymakers and policy practitioners can advise on what kind of data are needed to inform decisions, how different kinds of data will ‘land’ or be interpreted, and what level of detail is useful in order ensure research and evidence briefings are read.

There are examples of such good practice, such as the collaborations between Brook (a UK-based charity providing wellbeing and sexual health support, information and services for under-25s) and academic researchers to provide evidence for and develop online training resources for teachers around consent, sexual pleasure, and abortion (with researchers from the University of Sussex and the Open University; see learn.brook.org.uk), and to deliver research on digital intimacies with practical recommendations for teachers and policymakers (see McGeeney and Hanson 2017). Produced with the NSPCC, Welsh Women’s Aid, the Children’s Commissioner for Wales and the Welsh Government, *AGENDA: a young people’s guide to making positive relationships matter* is an online toolkit co-produced with young people, which supports them to speak out about and back to gender and sexual injustices at both primary and secondary school levels (Renold 2016, 2019). AGENDA comprises a collection of practical and creative ways for young people to explore and voice their feelings, engage in issues that matter to them, and challenge stereotypes and norms. The resource has had a wide impact; it reached 1,400 young people, 1,000 practitioners, 500 teachers, and 100 academics in the first 12 months since its publication and its activities and principles have been embedded into the provision of RSE in Wales (ESRC 2018). Another example of collaborative academic research and youth-led advocacy has involved research on homophobic and transphobic bullying in Europe (Formby 2013) to develop resources for practitioners by the International Lesbian, Gay, Bisexual, Transgender, Queer, and Intersex Youth and Student Organisation, including a short film about the research and subsequent minimum standards to combat homophobic and transphobic bullying (IGLYO 2013). This guidance was launched at the European Parliament in 2014 and has gone on to inform further work on inclusive education practices in Europe.

Working across sectors and across disciplines presents challenges as well as opportunities. Participants noted difficulties posed by working across boundaries, and in aligning multiple, sometimes conflicting, agendas. For example, a research project across the fields of social psychology, media studies, and gender studies found that researchers from different disciplines had different ideas about what constitutes healthy sexual development (Litsou and Byron 2019). There may also be challenges in reconciling conflicting requirements of the health and education sectors for research that is useful and can inform practice, and the need for academics to have the freedom to identify interesting areas of enquiry which may not directly generate evidence of use to a sector or organisation. In addition, some practitioners or organisations may face political constraints on the extent to which they can engage with certain ideas or recommendations, with one workshop participant noting that it would be unlikely that the government would promote the importance of pleasure in its RSE guidance. Participants raised practical constraints to collaboration; practitioners are likely to be willing collaborators in research projects but may be prevented from doing so because of monetary and time constraints: research budgets should cover this. There can also be practical challenges for academics, for example an organisation seeking an academic partner may have a very limited budget, but also require that the project be completed in a shorter timescale than that required to obtain funds from most potential sources of research funding.

### Combine long*-*term and short-term projects

Research must be able to respond to government and policy agendas in order that policy is based on high-quality evidence. This requires the academic sector to be able to produce a swift response when evidence is needed, make skills available to other sectors at a speed that the sector needs, and present the resulting research in a way that is easy to understand but not at the expense of accuracy (Oliver and Cairney 2019). It also requires that research users seek out research evidence. A rapid response is facilitated by relationship building between researchers and decision-makers, so that researchers are available and accessible when research is needed (Oliver and Cairney 2019), and research users are up to date on current evidence and gaps. A practical action to facilitate this would be through a research-into-practice group or expert meeting including academics, policymakers and practitioners in digital intimacies and RSE which meets regularly (e.g. twice a year) to discuss (a) how to translate existing research into practice and (b) current challenges or gaps in understanding of effective practice which can inform research. A supportive and collaborative group might enable policymakers and practitioners to be more open about what they do not know and academics to be closer to the reality of policy and practice everyday implementation. A key limitation to swift responses from academic researchers is the precarity and heavy workload of many academic jobs, with their corresponding absence of salaried free time that could be used for these types of thoughtful, rapid contributions which are not usually valued in academic recruitment and promotion. The problem of precarity is not limited to academics, professionals working with young people labour under heavy workloads and uncertain funding.

Some policymakers and practitioners at the workshop suggested that the strongest contribution academic researchers could make to strengthening the process from evidence to practice would be through working to shorter time frames, producing reports and reviews on a quicker turnaround than usual academic timeframes, reviewing literatures, doing meta-analysis, and offering support to research projects led outside the university, where appropriate. This is useful and important work but cannot represent the entirety of academic research. Academics must not be constrained by – or beholden to – government and policy agendas and short-term projects. Not only is academic research of value in identifying important questions that will not have occurred to policymakers, but research agendas should not be solely driven by the policy agenda. Conducting useful, meaningful research often requires engaging with different audiences and beneficiaries and learning from a range of academic and non-academic literature from multiple disciplines. Research must also explore new lines of enquiry; challenge received wisdom; challenge harmful, non-evidence-based or ineffectual strategies and agendas; and recognise and advocate for the value of longer and slower, co-productive and participatory research processes (Miles, Renedo, and Marston 2018; Collin and Swist 2016; Selwyn and Stirling 2016). There is value both in research that can meet both shorter-term needs for evidence and understanding, and research that addresses longer-term questions. Short- and long-term projects can then be brought together to build up a picture that incorporates both broader, more comprehensive understandings of young people and digital intimacies and seeks to answer specific, policy-driven questions.

### Build research around an ethical framework and interrogate concepts of pleasure and harm

Since much current research and practice (although by no means all of either), focuses on the risks and harms of technology and sexual life (Flood 2009; Brown and L’Engle 2009), it is important that both also interrogate concepts of risks and harm, and recognise that these narratives can in themselves be harmful (Lerum and Dworkin 2009). For example, the approach of research and campaigns that frame sexually explicit image sharing (often referred to as ‘sexting’) as a risky activity that individuals must bear responsibility for constructs girls as both victims of image-sharing related bullying and as responsible for preventing it (by abstaining). Such framing can be harmful because by normalising the idea that there are different risks and responsibilities for boys and girls it reinforces stereotypical and heterosexualised gender norms and shifts responsibility from perpetrators of unethical behaviour onto victims (Dobson 2018).

It is possible to frame research projects around questions of ethics rather than risk and harm. Building research around an ethical framework is essential to producing high quality, relevant research on young people and digital intimacies, and may be a productive way of critiquing the harm narrative and an effective means of redirecting the conversation away from risk and harm and towards providing a supportive space for young people. This would be facilitated by an approach that starts with a blank sheet and elicits from young people themselves how they and their peers experience digital intimacy and their perceptions of the risks, benefits, harms, mitigations and solutions, as opposed to one that premises a conversation on assumed harms and asks young people for solutions. Considering carefully whether the measured outcomes might stigmatise or negatively frame some behaviours, and if so whether they can be measured in a more balanced way, avoiding making assumptions about problems or harms, will also avoid the bias induced by such assumptions. For example, if a study starts by assuming that certain behaviours are harmful, the results will be biased because the data collected may not allow any evidence to the contrary, and because participants may gauge the viewpoint of the researchers and be unwilling to disclose contradictory information for fear of being judged, or may not participate in the research at all. Ethics centred approaches have been productively translated into practice; Carmody’s sexual ethics framework for example has been used by researchers and practitioners to inform RSE (Carmody 2008).

### Start with what good looks like

Often, ‘healthy sex’ is promoted as a goal that can be achieved by minimising risks, conforming to socially normative relationships, and abstaining from or limiting encounters with sexual media (Allen and Rasmussen 2017). For example, in the public health and psychology literatures, having multiple partners is sometimes considered a ‘problematic’ behaviour associated with negative outcomes because of higher rates of STI acquisition among those with multiple partners (Vasilenko and Lanza 2014; Grant, Lust, and Chamberlain 2019). This ignores individual motivations and what might be gained from having multiple partners, and it is heteronormative, excluding young people oriented towards polyamory or other forms of queer relationships. More recent policy approaches to pornography consumption among young people sit decidedly on the side of regulatory mechanisms that seek to limit access, including the delayed (now shelved) attempt to introduce age-verification for porn sites (BBFC 2018). Focussing on disease, dysfunction, risk and harm limits understanding and opportunities for holistic RSE. Instead of how to protect young people, the focus of research could shift to what ‘good’ sex and relationships would look like for a young person to identify and build the assets^2^ people need to reach this goal. McKee at al. (2010) have considered this by setting out fifteen domains of Healthy Sexual Development as part of a multidisciplinary framework. These domains include an understanding of safety; freedom from unwanted sexual activity; agency; an understanding of consent and ethical conduct; awareness and acceptance that sex is pleasurable; and competence in mediated sexuality.

In research on young people and digital intimacies, establishing a consensus with young people on what would characterise ‘good’ sex and relationships might refocus the discussion on autonomy, consent, communication, diversity, pleasure, equality, and inclusion. It also allows for a nuanced discussion of the trade-offs that young people face in their decision-making around engagement with digital intimacies, and the structural factors that facilitate and constrain their behaviour (Hendry 2017; Fu 2018). For example, Ringrose et al. (2013) examined the gender sexual double standards that shaped participation in and experiences of image sharing, showing how young men and women experience differential consequences of sharing sexually explicit images, and showed how image sharing can be a useful or empowering way of negotiating consensual, ‘safe’, and wanted sex or intimacy and of identifying likes and dislikes.

Research from within media and cultural studies, sociology, gender and sexuality studies has sought to explore the importance for young people of being online. Waite (2011) argues that social media interactions give young people a sense of belonging, making friendships visible in spaces that are perceived as relatively safe. Making sophisticated judgements about privacy and safety, reflected in the information they choose to share online (Livingstone 2008), young people are continually developing ‘tacit rules and understandings’ (Pangrazio 2019) through their participation on social media so it is counterproductive to insist that adults know best. Messaging in particular plays a key role in maintaining everyday relationships, and mobile technologies are valued because their immediacy increases intimacy (Lasen 2004). For young people, technology enables relationships. For LGBT+ young people, in particular, online communications counter the potential isolation and stigma experienced in offline spaces (DeHaan et al. 2013; McGeeney and Hanson 2017). This is not to suggest that online activities are not without risks but to stress that interventions must recognise young people’s agency in, commitments to, and rewards in, self-expression and sexual development.

An approach based on what good looks like has implications for influencing practice. For example, the Healthy Sexual Development framework has been endorsed and used by practitioners working with young people, while Brook’s *Sexual Behaviours Traffic Light Tool* (www.brook.org.uk/our-work/category/sexual-behaviours-traffic-light-tool)^3^ supports professionals working with children and young people to identify and respond to sexual behaviours, including by understanding and distinguishing healthy sexual development from harmful behaviour. To ensure they meet young people’s needs, future initiatives could include forms of digital literacy which start from young peoples’ experiences (positive and negative), interests (sexual and non-sexual), and existing skills in navigating and producing digital content.

### Be inclusive in all stages of research

Inclusivity is important in terms of gender and gender identity, sexual identity, religion, class (dis)ability, and ethnicity. Inclusive research should recognise the ways in which young people’s experiences of digital intimacies are diverse and shaped by intersecting inequalities. Particularly in sexual health research, a heteronormative approach prevails. For example, sex is often defined as involving penetration with a penis (Ansara 2015), and minority groups in terms of sexual identity and gender identity are often treated as a homogenous group or excluded from analyses altogether because of sample size limitations (Carrotte et al. 2016). Heteronormativity is a barrier to accessing information and services and is reinforced by a lack of inclusivity in research. Another key area is inclusivity with regard to Special Educational Needs and Disability (SEND), to ensure that research recognises both universal rights to sexuality of young people with SEND, and the diverse challenges and specific vulnerabilities they may face. Research that does not take a sufficiently inclusive approach can be frustrating for participants who may not feel able to express their views or experiences adequately, or who may even feel misrepresented (Carrotte et al. 2016). It may also frustrate and exclude those reading outputs of research, who may feel they do not ‘fit’ the assumptions of the researchers or that their experiences are not represented (Carrotte et al. 2016).

Several groups have published recommendations for increasing inclusivity in research, including providing multiple answer categories to questions about, for example, sex, gender and sexual identity; using culturally appropriate language that does not cast certain identities or practices as ‘normal’; appropriately contextualising the research (by acknowledging the limitations of the study sample) and avoiding overgeneralising the findings (by recognising that the ‘general’ population is a diverse population and that minority groups are part of the general population) (Goins and Pye 2013; Ansara 2015; Carrotte et al. 2016). Researchers should resist assuming homogeneity within ‘minority’ groups and avoid reinforcing stereotyping (Kneale et al. 2019).

## Priority research topics

Alongside these fundamental approaches to doing research with young people and digital intimacies to inform RSE, we identified a number of priorities for the content of research.

### Everyone needs lifelong relationships and sex education

Participants had a wide range of expertise and this resulted in discussions that covered a diversity of young people and RSE settings. There was a consensus that RSE is a universal need, which begins at a young age. However, there is relatively little research into RSE provision for younger children. Learning about sex and relationships is something that continues into adulthood (McKee et al. 2010), and parents and caregivers, too, want to know how they can be talking about sex and relationships with their children (Turnbull, Van Wersch, and Van Schaik. 2008). Researchers could investigate how to identify and address the most salient issues with regards to digital intimacies and young people among parents and caregivers, to engage them in young people’s RSE learning. Related to this is a move away from conceptualising issues around digital intimacies as specific to young people – many themes in this area are applicable at all ages.

Alongside the universal need for high-quality RSE, many groups of young people have specific needs. Previously, we highlighted the necessity for research to be inclusive in its approach; here we stress that it must also be inclusive in its content. Research that investigates the specific needs of young people based on their gender identity, sexual identity, ethnic group, religion, or (dis)ability (including SEND) will help ensure that RSE is relevant to and inclusive of all young people, as well as inform the development of resources to support young people with different needs. By addressing intersecting vulnerabilities, RSE also has the potential to address inequalities.

Some young people may be more vulnerable than others to potential harms related to digital intimacies. For example, young LGBT+ people may face more risks when connecting with others online, due to greater isolation (particularly in smaller communities) and stigma (McGeeney and Hanson 2017), while simultaneously digital spaces may offer them important connection and resources so that protection and/or prevention are not necessarily appropriate responses to recognising potential vulnerability. Young LGBT+ people have identified that education related to online safety does not speak to them when it only focuses on ‘risks’ and ‘dangers’ rather than also potential feelings of safety and happiness stemming from identity affirmation and/or a sense of belonging and community online (Formby 2017; Hatchel, Subrahmanyam, and Birkett 2016). This may also apply to other groups of young people. Research that explores who might be particularly vulnerable, in which contexts and why, will help understand how social inequalities shape young people’s sexual practices and experiences. This will also help identify where targeted approaches might be effective.

### Relationships, including friendships

The group discussed the value of moving towards discussions that go beyond sexual relationships. Intimacy is not limited to sexual relationships; young people express intimacy and take part in intimate practices through friendships too, and how these are digitally mediated has received less attention in the literature (see Berriman and Thomson 2014; Setty 2018; Jaynes 2019 for exceptions). Research that explores friendships and relationships will shed light on the functions that digital intimacies play in the contexts of identities, friendships and peer groups. It also has potential to situate digital intimacies in a broader context than ‘sexual intimacy’, allowing nuanced conceptualisations of the meanings of digital intimacies for young people and facilitating a move away from considering digital intimacies as distinct, definable practices. It would be helpful to young people identifying as asexual or aromantic who want to access feelings of connection and intimacy without it being assumed this relates to sexual attraction or desire. Research that considers digital intimacies in the context of friendships can facilitate research with children. For example, Renold and colleagues have used creative and participatory methods to explore peer cultures and friendship groups, and how gender, sexuality and consent map onto these, in primary and secondary schools (Renold 2016).

### Every day, rather than problematic engagement with digital intimacies

Although there is great diversity in scholarship on young people’s engagement with digital intimacies, much of the research that reaches decision-makers and is translated into policy considers the practices of pornography, ‘sexting’, or both (Barrense-Dias et al. 2017; Horvath et al. 2013; Peter and Valkenburg 2010). However, focusing on discrete practices does not recognise the complexity of young people’s engagement with digital intimacies. Furthermore, the rationale for much of this research is not to understand young people’s motivations and behaviours but to identify the harmful outcomes of these behaviours. Research that examines the functions that digital intimacies play in young people’s day to day experiences would help contextualise young people’s digital practices and situate them in everyday life. For example, Thomson, Berriman, and Bragg (2018) explore how children engage with technology in their everyday lives, and show how their engagement is shaped by their experiences at home and at school. For LGBT+ young people particularly, the digital world can offer important sites of identity experimentation and/or control (Jenzen 2017).

A broader conceptualisation of digital practices and an examination of how they are used may also facilitate a move away from focussing on discrete ‘hot topic issues’ towards framing research around deeper, broader themes. From a practice perspective, the *DO … RSE* (https://www.dosreforschools.com/) approach does this by putting young people in charge of their own learning, recasting the practitioner from an expert delivering knowledge to a facilitator supporting young people to broaden their investigation in a specific area. This approach positions young people as experts in their own lives and needs.

### Motivations, risks, trade-offs and rewards

Focusing on harmful outcomes results in a lack of understanding about young people’s motivations for engaging with digital intimacies, and how they negotiate the trade-offs between risks and rewards. After all, even if digital intimacies were harmful, they must come with perceived rewards that outweigh the risks for those young people who choose to participate. For example, McGeeney and Hanson (2017) describe how for some LGBT+ young people, leaving privacy settings open was seen as necessary to meet people, owing to the small numbers of out LGBT+ young people living in their communities. In other research LGBT+ young people identified the benefits (in the absence of LGBT-inclusive sex and relationships education) of interaction with (LGBT+) strangers to learn from their experiences (Formby and Donovan 2020). In Madell and Muncer’s (2007) research on young people’s use of mobile phones, respondents talked about the ways they used technology to manage their emotions and facilitate communication about difficult subjects or during difficult times. A better understanding of these trade-offs will help inform RSE that can support young people to navigate digital intimacies in their lives. Moreover, young people are using digital media to seek information in myriad ways. Digital technologies provide alternative informal sources of sexuality which young people often find more engaging and perceive as more relevant than formal sexuality education (Abidin 2017; Makleff et al. 2019). Technology allows young people to seek out and access information about sex autonomously and independently (Ragonese, Bowman, and Tolman 2017) and to share and produce that information amongst themselves. These studies highlight the opportunities digital media may offer for practitioners to harness channels that are relevant, engaging and meaningful to young people.

## Conclusion

The introduction of statutory RSE in schools in England has focused attention on the need to improve understandings of young people’s engagement with digital intimacies both for its own sake, and to inform the development of RSE. The government has committed to reviewing the guidance on RSE within three years from first required teaching and every three years after that point (Department for Education 2019), providing a continuing opportunity for research to inform policy. For statutory RSE to have the desired impact, delivery must be evidence-based and relevant to young people’s lived experiences. However, while the guidance highlights the importance of addressing topics like pornography, ‘sexting’, and use of online media, there is no accompanying guidance on how these issues should be addressed. We call for more research that is informed by the fundamental approaches outlined in this paper – work that puts young people at its centre; foregrounds inclusivity; and allows a nuanced discussion of pleasures, harms, risks and rewards, which can be used by those working with young people and those developing policy. Generating such research is likely to be facilitated by participation, collaboration and communication with beneficiaries, between disciplines and across sectors. Taking such an approach, academic researchers, practitioners and policymakers agree that we need a better understanding of RSE’s place in lifelong learning, which seeks to understand the needs of particular groups, is concerned with non-sexual friendships, and does not see digital intimacies as disconnected from offline everyday ‘reality’. As academic research asks these questions using the approaches we have outlined, it will continue to make a valuable contribution to the expansion of a strong evidence-base – that is properly informed by young people’s voices and experiences – to inform the development and delivery of RSE that meets young people’s needs both in and out of school settings, in the UK and around the world.

## Supplementary Material

Agenda_-_Young_people__digital_intimacies_and_sex_and_relationships_education.doc

## Notes

1. The term ‘sexting’, a portmanteau of ‘sex’ and ‘texting’, is often used as a catch-all term for the creation and sharing of nude, semi-nude and sexually explicit imagery via digital means, and is the term used in the Department for Education guidance. While the term ‘sexting’ is often used in mainstream media, and in research, policy and practitioner discourses, this language has been found not to reflect young people’s practices and experience, nor the language that they themselves use (Albury et al. 2013; Setty 2018).

2. An assets-based approach (see Morgan and Ziglio 2007) is gradually gaining traction in policy development. Identifying the assets and critical skills children and young people need for developing and managing intimate relationships and appraising harms and benefits would not only help inform effective programmes but could also be used to evaluate their impact.

3. The development of the *Traffic Lights Tool* was funded by the UK government’s Department for Education to promote the safeguarding of young people.
