# Supplementary material for: What and how: doing good research with young people, digital intimacies, and relationships and sex education
Source: Sex Educ. 2020 Mar 17;20(6):675–91. doi: 10.1080/14681811.2020.1732337 (PMC7872220; doi:10.1080/14681811.2020.1732337)
Supplement: Agenda_-_Young_people__digital_intimacies_and_sex_and_relationships_education.doc [file CSED_A_1732337_SM7485.doc]

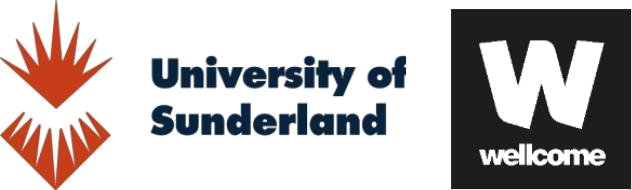


**Workshop to discuss the future research agenda on young people,**

**digital intimacies, and sex and relationships education**

**Background**

School based sex and relationships education will become compulsory in England in 2019. Topics like pornography, sexting and use of online media will be included but the form that this will take is unclear. With changes in technology have come developments in the ways in which individuals can communicate and connect, as well as new opportunities for engaging in intimate practices using technologies. Digital intimacies can be considered as encompassing a wide range of practices, including producing, sharing, broadcasting and viewing intimate content. These might include sexting; taking and sharing selfies; using hook-up apps; communicating about sex and relationships; searching for information and advice; and creating, accessing and circulating sexual content online, through social media and through apps.

**Objective**

The aim of this workshop is to bring together individuals from research, practice and policy backgrounds **to advance the future research agenda on young people, digital intimacies, and sex education.**

**Approach**

Through presentations and discussion, we anticipate that the workshop will generate new conversations and new ideas, and be an occasion to build networks between a wide range stakeholders working in this area. We hope it will lead to new conversations between research, practice and policy sectors to identify new possibilities of thinking about young people and digital intimacies.

The discussion will be flexible, and led by questions arising out of the meeting. Topics discussed might include:

- What do participants working in policy and practice consider to be the main questions that would help improve services?
- How might future research respond to practice and policy questions?
- How can researchers, practitioners and policy-makers collaborate to better understand the main issues facing young people with regard to digital intimacies, and develop helpful and effective interventions and resources?
- What are the obstacles to implementing good resources?
- How can different disciplines work together to build a more complete and complex picture of how young people navigate, understand, consume, react to and engage with digital intimacies?
- How can research involve young people themselves in an active and participatory way?
- What are the challenges to doing research with young people and digital intimacies?

**Expected outcomes**

A set of research priorities, to be written up as a commentary for publication or short report.

**
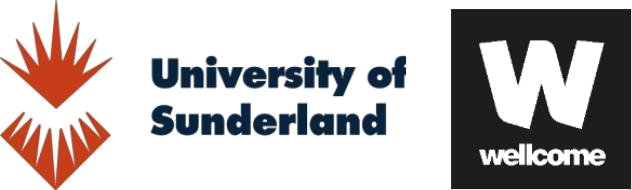
**

**Date:** Friday 22nd June 2018

**Time:** 09.30 - 16.00

**Location:** Marjorie Sykes Room, Friends House, 173-177 Euston Road, London

**AGENDA**

09.30-10.00 Tea, coffee, pastries

10.00-10.05 **Welcome**

*Clarissa Smith; University of Sunderland*

10.05-10.15 **What is this project about?**

*Rachel Scott; University of Sunderland*

Presentation

10.15-11.15 **Who we are, what we do, why we’re here**

Short introductions from all participants

11.15-11.30 Break

11.30-11.40 **Sex and relationships education – setting the scene**

*Justin Hancock; BISH*

Presentation

11.40-12.30 **Doing research across disciplines and across sectors – challenges and opportunities**

*Alan McKee; University of Technology Sydney*

Presentation and discussion

12.30-13.15 Lunch

13.15-14.30 **Small group discussions** **and feedback**

14.30-14.45 Break

14.45-15.45 **Roundtable discussion**

*Chair: John Mercer; Birmingham City University*

Priorities for advancing the future research agenda on young people, digital intimacies and sex and relationships education

15.45-16.00 **Summary and close**

*Feona Attwood; Middlesex University*

***
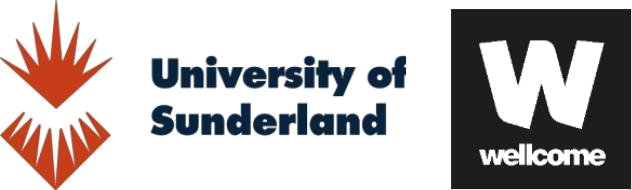
***
